# YKL-40 as a possible marker of neutrophilic asthma

**DOI:** 10.3389/fmed.2023.1115938

**Published:** 2023-02-08

**Authors:** Krzysztof Specjalski, Jan Romantowski, Marek Niedoszytko

**Affiliations:** Department of Allergology, Medical University of Gdańsk, Gdańsk, Poland

**Keywords:** YKL-40, chitinase-like protein, asthma, asthma biomarker, neutrophilic asthma

## Abstract

Asthma is a heterogeneous chronic disorder of the airways, with inflammation and bronchial hyperresponsiveness as its major underlying phenomena. Asthmatics vary in terms of inflammation pattern, concomitant pathologies, and factors aggravating the course of the disease. As a result, there is a need for sensitive and specific biomarkers that could facilitate diagnosing asthma as well as phenotyping in everyday practice. Chitinases and chitinase-like proteins (CLPs) seem promising in this field. Chitinases are evolutionarily conserved hydrolases that degrade chitin. In contrast, CLPs bind chitin but do not have degrading activity. Mammalian chitinases and CLPs are produced by neutrophils, monocytes, and macrophages in response to parasitic or fungal infections. Recently, several questions have been raised about their role in chronic airway inflammation. Several studies demonstrated that overexpression of CLP YKL-40 was associated with asthma. Moreover, it correlated with exacerbation rate, therapy resistance, poor control of symptoms, and, inversely, with FEV_1_. YKL-40 facilitated allergen sensitization and IgE production. Its concentration was elevated in bronchoalveolar lavage fluid after an allergen challenge. It was also found to promote the proliferation of bronchial smooth muscle cells and correlate with subepithelial membrane thickness. Thus, it may be involved in bronchial remodeling. Associations between YKL-40 and particular asthma phenotypes remain unclear. Some studies showed that YKL-40 correlates with blood eosinophilia and FeNO, suggesting a role in T2-high inflammation. Quite the opposite, cluster analyses revealed the highest upregulation in severe neutrophilic asthma and obesity-associated asthma. The main limitation in the practical application of YKL-40 as a biomarker is its low specificity. High serum levels of YKL-40 were also found in COPD and several malignancies, in addition to infectious and autoimmune diseases. To conclude, the level of YKL-40 correlates with asthma and some clinical features in the whole asthmatic population. The highest levels are found in neutrophilic and obesity-related phenotypes. However, due to its low specificity, the practical application of YKL-40 remains uncertain but could be useful in phenotyping, especially when combined with other biomarkers.

## Introduction

Asthma is an airway disorder with chronic inflammation and bronchial hyperresponsiveness as its major underlying phenomena. They lead to transient episodes of breathlessness, coughing, wheezing, and chest tightness. In fact, asthmatics are a heterogeneous population, and, as a consequence, asthma is an umbrella term describing a clinical picture rather than a single pathologic process. For this reason, asthma is diagnosed on the basis of symptoms reported and the demonstration of variable bronchoconstriction over time (1).

The concepts of extrinsic and intrinsic asthma have been introduced several decades ago. The former is atopic and usually manifests early in life. The latter develops in adults, more often in women, and is not associated with any allergy (2). This distinction had great significance because only in some patients did airborne allergens cause asthma exacerbations, and interventions based on allergen avoidance have proved beneficial. However, this division did not fully reflect the type of inflammation and had implications for pharmacotherapy. Therefore, it has been abandoned (3). More recently, concepts of subtypes of asthma have been widely discussed once again, with several proposals for phenotypes (observable properties of an organism produced by interactions of the genotype and the environment) and endotypes (pathological pathways explaining properties of phenotype) (4, 5). Cluster analyses revealed that age of asthma onset, atopy, gender, obesity, sputum eosinophilia, or neutrophilia are among the variables strongly differentiating asthmatics. Thus, the following phenotypes have been proposed: T2-high related: early onset allergic, late-onset eosinophilic, exercise-induced, and T2-low related: obesity-related, neutrophilic, etc. (6).

In the era of biological therapy, phenotyping has been recommended in every case of severe asthma (1). Phenotype-tailored therapy requires thorough enrollment based on clinical characteristics in addition to biomarkers in order to distinguish between potentially good and poor responders. Sputum eosinophils and FeNO are considered good biomarkers of eosinophilic asthma as they correlate with clinical outcomes and therapy with inhaled steroids. Atopic asthma is associated with elevated serum total IgE (7, 8). In contrast to eosinophilic asthma, phenotypes not related to T2-high inflammation have not been precisely defined. Little is also known about their immunological and inflammatory determinants. As a consequence, no biomarkers have been introduced into clinical practice to date.

As there are numerous unmet objectives in the diagnosis and phenotyping of asthma, it is necessary to search for novel biomarkers. They could be useful in diagnosing asthma, monitoring its course, evaluating the severity of inflammation and treatment efficacy. Finally, they could enable phenotyping and endotyping with the use of easily available and repetitive tools. Over the last 15 years, dozens of studies have been published on the possible application of chitinases and chitinase-like proteins in this field.

Chitin is, after cellulose, the second most abundant polysaccharide in nature. It is a significant component of fungal cell walls, nematode sheaths, and the exoskeletons of several animals (crustaceans, insects, and arachnids). Chitinases are evolutionarily conserved hydrolases. By degrading chitin, they play a crucial role in the homeostasis of several organisms. Chitinase-like proteins (CLPs) lack enzymatic activity due to amino acid substitutions taking place in the process of evolution in their active site (9). Despite this, they have retained active-site carbohydrate binding and have been named chi-lectins. The physiological role of binding chitin by CLPs remains enigmatic. It is hypothesized that CLPs alter immune response to chitin and its fragments (10). Because mammals do not synthesize chitin, until recently it was assumed that chitinases were not produced either. Nowadays, it is believed that they mainly play a role in response to parasitic or fungal infections (11). However, in lower life forms, they also control inflammation, tissue repair, and remodeling.

Mammalian chitinases and CLPs (acidic mammalian chitinase–AMC; oviductin; chitotriosidase; human cartilage glycoprotein–HcGP/YKL-40) are produced by neutrophils, monocytes, macrophages, cultured chondrocytes, and synovial cells. The best known of them, YKL-40, was first described in mouse breast cancer cells as BRP-39. Both murine BRP-39 and its human analog, YKL-40, are products of genes located on chromosome 1 (the CH3L1 gene in humans). YKL-40 has been extensively investigated in recent years. Its elevated serum levels were demonstrated in obstructive diseases, autoimmune disorders, several types of cancer, and during myocardial infarction (12–14). The role of CLPs in the pathogenesis of these diseases is not fully understood. It has been suggested that YKL-40 regulates vascular endothelial growth factor (VEGF), which is involved in inflammation and angiogenesis (15, 16). In asthma, VEGF enhances the proliferation of bronchial smooth muscles and mucus hypersecretion (17, 18). Both in the general population and in patients suffering from chronic diseases, an elevated serum YKL-40 level is associated with increased mortality (19, 20). In cancer, high levels often correlate with a poor prognosis and the presence of metastases (21, 22).

It has been demonstrated that silencing the YKL-40 gene can inhibit the occurrence of inflammation by lowering the concentrations of IL-8 and MMP-9, in addition to the migration and invasion of cancer cells (23). On the other hand, there are significant discrepancies in the results of studies attempting to find correlations between YKL-40 and traditional inflammatory markers. Some of them demonstrate correlations with CRP and IL-6, while others do not. It can be explained by the heterogeneity of studied populations and diseases (24–26).

## YKL-40 as a marker of asthma

In the past 15 years, dozens of studies have been published comparing serum concentrations of YKL-40 between asthmatics and healthy controls. Recently, two meta-analyses summarized their results. The one by Jin et al. was based on 17 articles involving 5,696 subjects. It revealed significantly higher serum levels of YKL-40 in asthmatics compared to controls, irrespective of age, gender, and race (27). Pan et al. analyzed 15 studies involving 1,647 asthmatics and 1,259 healthy controls. It confirmed higher levels of YKL-40 in both adult and pediatric asthmatic populations compared to healthy controls (28). Although overexpression of YKL-40 is well-documented and this protein was proposed as a novel biomarker for this disease, its low specificity can limit its use in this capacity. So far, no study has evaluated its sensitivity and specificity in relation to the current GINA standard for diagnosing asthma (1). The available papers compared YKL-40 levels between asthmatics and healthy controls, as opposed to the general population with several possible concomitant diseases.

Moreover, there is currently no consensus on reference values for YKL-40 serum concentrations. In a group of 3,130 participants recruited from the Danish general population between the ages of 20 and 80 with no known chronic disease, the median plasma YKL-40 was 40 μg/l (2.5–97.5% reference levels: 14–155 μg/l) with no gender differences. A 10-year follow-up revealed that YKL-40 increased exponentially with age (mean increase: 1.5 μg/l/year) (19). In contrast, in another study, the median YKL-40 serum level was 80 μg/l in healthy children and 102 μg/l in healthy adults, with no age differences in subjects younger than 70 years (29).

In conclusion, although an elevated level of serum YKL-40 is strongly associated with asthma, its low specificity seems to be a major obstacle to its application in everyday practice.

## Role of YKL-40 in the mechanism of asthma

Chitinases and CLPs play a role in the immune response to parasitic infection and are overexpressed in inflammation sites and remodeling processes. Human plasma levels of CLPs are elevated, e.g., in malaria, fungal, and helminth infections. Additionally, *in vitro* cell culture experiments show an increase in their expression when macrophages or neutrophils are stimulated with IFN-γ, TNF-α, or toll-like receptor ligands (30). In murine models of parasitic infections, CLPs are secreted in response to IL-4 by alternatively activated macrophages believed to resolve inflammation and lead to wound healing by enhancing fibronectin and TGF-β secretion, extracellular matrix deposition, and angiogenesis (15, 16, 31).

It is well known that a T2-high anti-parasitic response shares some key features with allergic inflammation, including secretion of IgE and cytokines (IL-4, IL-5, and IL-13), in addition to eosinophil infiltrations (32). Thus, it is believed that the mechanisms that originally evolved to deal with parasitic infections may lead, when poorly controlled, to atopic diseases. It is also hypothesized that chitinases and CLPs are simply other inflammatory mediators previously associated with an antiparasitic defense that are activated by allergic inflammation (11).

Relations between chitinases and atopy have been investigated several times. BRP-39, the murine homolog of human YKL-40, was found to facilitate allergen sensitization and IgE production. Its concentration in bronchoalveolar lavage fluid increased after an allergen challenge. Mice with BRP-39 null mutations (BRP-39 -/-) did not respond to ovalbumin sensitization with IgE secretion. Concentrations of IL-4 and IL-13 were significantly reduced in their lung tissue compared to wild-type mice (33). In the murine model of asthma, acidic mammalian chitinase was upregulated, and its expression was induced by Th2 cytokines: IL-4 and IL-13. On the contrary, inhibition of AMC with the use of anti-AMC sera led to decreased Th2 inflammation and prevented airway hyperresponsiveness in the ovalbumin challenge (34). AMC is now believed to be a crucial determinant of IL-13 effector response activation (11). Murine CLP YM-1 is a potent chemotactic agent for eosinophils and Th2 cells (35).

The effect of chitin itself on immune responses in the lungs is still controversial. In an animal model, oral administration of chitin facilitated the resolution of T2-high inflammation (36). On the contrary, more recent studies demonstrated that the administration of chitin into the lungs resulted in the accumulation of eosinophils, neutrophils, and macrophages in the tissue and their increased number in BAL fluid (37).

As YKL-40 induces the proliferation of mesenchymal cells, migration, and adhesion of vascular smooth muscles, it has been hypothesized that this could also play a role in bronchial remodeling in asthma (38). This is in line with the finding that serum and bronchial lavage levels of YKL-40 correlate with subepithelial membrane thickness (39). Bara et al. found that *in vitro*, YKL-40 increased bronchial smooth muscle (BSM) cell proliferation and migration, which are two of the characteristics of bronchial remodeling. Moreover, its epithelial expression correlated with the mass of BSMs (40).

## Relations between YKL-40 concentration and the clinical course of asthma

It has been demonstrated that in asthmatics, serum YKL-40 levels correlate with severity and the presence of exacerbation and reversely correlate with FEV_1_ (25, 41). In some studies, correlations with blood eosinophilia and FeNO have also been observed, which may suggest some role for CLPs in T2-high inflammation (41, 42).

In the prospective study of Lai et al. 103 newly diagnosed asthmatics were evaluated over the course of 8 weeks of inhaled corticosteroid (ICS) treatment. It was confirmed that the initial level of YKL-40 had an inverse correlation with FEV_1_ and symptom severity as measured by the use of an asthma control test (ACT). Serum levels of YKL-40 decreased significantly after the introduction of treatment. Interestingly, the dose of ICS necessary to control asthma correlated with the initial YKL-40 level. Thus, it seems that YKL-40 correlates with inflammation intensity and could predict response to treatment (43). Very similar results were found after long-term prospective observation of patients with a longer history of asthma. In participants with stable asthma monitored over 12 months, YKL-40 serum levels were associated with an increased risk of moderate-to-severe asthma exacerbations. Moreover, YKL-40 was found to be an independent biomarker of negative responses to anti-asthma therapies (44). In contrast, YKL-40, just like the well-established biomarkers (periostin, osteopontin), was not useful in predicting the future development of asthma in preschool children with recurrent wheezing (45).

Levels of YKL-40 are affected by genetic predisposition. It was demonstrated that some single nucleotide polymorphisms (SNPs) in the CH13L1 gene are associated with higher serum levels of this protein in addition to the clinical characteristics of their carriers. The GG genotype in rs4950928 is related to lower YKL-40 secretion and higher FEV_1_ values, whereas the GG genotype in rs12141494 has protective properties in relation to severity and is associated with higher post-bronchodilator FEV_1_ values and lower YKL-40 levels (42). Other polymorphic variants have been linked to an increased risk of asthma and atopy (46, 47).

## YKL-40 in distinct phenotypes of asthma

A large body of evidence appeared to confirm the link between chitinases, CLPs, and T2-high inflammation. This was consistent with some studies finding the highest levels of YKL-40 in uncontrolled asthma and its correlation with blood eosinophilia or FeNO (41, 42). As a consequence, it was even speculated that YKL-40 could be considered a marker of eosinophilic asthma. However, in the following years, cluster analyses were conducted in “real-world” settings on quite substantial subgroups of asthmatics, grouping patients with similar clinical and molecular features of asthma. They revealed more complicated relations between YKL-40 and inflammatory phenotypes and disproved previous hypotheses on the close relationship with T2-high response.

Firstly, in an analysis by Ilmarinen et al. YKL-40 was significantly overexpressed in the “obesity-related asthma” and “smokers’ asthma” clusters compared to the “atopic asthma” cluster (48). This is in line with an earlier study by Specjalski et al. demonstrating that the mean level of YKL-40 was the highest in the subgroup of obese asthmatics (49).

It is generally acknowledged that obesity has a substantial impact on asthma. Obese asthmatics achieve good control of their asthma less frequently, experience exacerbations more often, and require higher doses of ICS (50). However, it is debatable whether obesity is a driving factor initiating asthma, a mere confounder, or just a frequent co-morbidity. Surely, obesity may enhance the perception of breathlessness in asthmatics due to greater energy expenditure while breathing. Moreover, it has been linked to chronic cough by causing gastroesophageal reflux. Obstructive sleep apnea, another pathology often associated with obesity, impairs the quality of sleep and leads to awakenings. Obesity is also strongly related to cardiovascular conditions. In contrast, in severe asthma requiring systemic steroids, obesity is a common side effect rather than the cause of the disease. Many authors link obesity with generalized inflammation characterized by elevated levels of TNFα, IL-6, and leptin (51, 52). A pro-inflammatory tendency in obese asthmatics, together with the concomitant diseases mentioned above and a relatively worse response to therapy, could be another explanation for high levels of YKL-40.

Secondly, in recent clinical studies, the YKL-40 serum level in asthmatics was found to correlate with neutrophil count, serum IL-6, and sputum IL-1β (44). In a cluster analysis by Gomez et al. the highest levels of YKL-40 were found in clusters characterized by an older asthma onset age, low total serum IgE, poor control of symptoms, frequent exacerbations, more severe airflow obstruction, and a high prevalence of obesity. These clusters had high neutrophilia in the induced sputum. Analysis of molecular differences in the sputum transcriptome revealed T2- low responses in these clusters. In contrast, T2-inflammatory enrichment was found in a cluster with better asthma control, low serum YKL-40 levels, and high total IgE (53).

**FIGURE 1:**
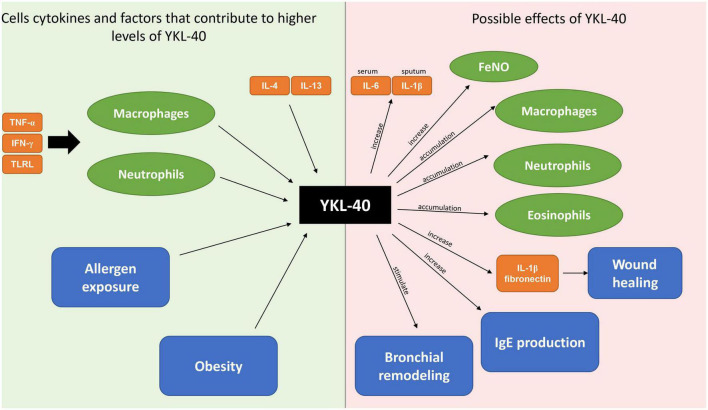
Origins and biological effects of YKL-40.

To conclude, YKL-40 may be considered a candidate biomarker for neutrophilic, obesity-related asthma (Figure 1).

## Role of YKL-40 in differentiating between asthma and COPD

Differentiating between asthma and COPD (chronic obstructive pulmonary disease) is a challenging issue in clinical settings, as signs and symptoms are largely shared by both entities. In fact, several patients with obstructive disease have some clinical characteristics of both asthma and COPD (asthma / COPD overlap; ACO) (1).

Comparative data on YKL-40 levels in asthma, COPD, and ACO is largely inconsistent. On the one hand, recent systematic reviews have demonstrated that YKL-40 is upregulated in both asthma and COPD patients compared to healthy controls (25, 26, 54). On the other hand, Sakazaki et al. found no significant difference in YKL-40 levels between patients with COPD and smokers without COPD (55). In another study, a population with COPD was found to be characterized by higher YKL-40 levels compared to asthmatics (56).

Some studies attempted to investigate the usefulness of combining several biomarkers in order to differentiate populations with asthma, COPD, and ACO. Evaluation of periostin and YKL-40 identified ACO with a sensitivity of 38% and specificity of 81%, whereas using eosinophil-derived endotoxin (EDN) with YKL-40 yielded a sensitivity of 45% and specificity of 82% respectively (57). Better results were achieved with the combination of YKL-40 and NGAL (Neutrophil Gelatinase-Associated Lipocalin). The highest levels of YKL-40 were noted in the COPD group, whereas NGAL was overexpressed in the COPD and ACO groups and did not differ between asthmatics and healthy controls. YKL-40 made it possible to distinguish patients with features of ACO from COPD with a sensitivity of 73% and a specificity of 68%. With NGAL, it was possible to distinguish ACO patients from the asthma group with a sensitivity of 93% and a specificity of 59% (58).

Thus, YKL-40 analyzed together with other well-established biomarkers seems to have better performance and could be used for differentiating between asthma, COPD, and ACO.

## Conclusion

Asthma is a heterogeneous disease with several phenotypes reflecting varied inflammatory patterns. One of the unmet goals is defining biomarkers to facilitate diagnosing and phenotyping asthma. It seems that chitinases or chitinase-like proteins are promising in this field. The most well-known of these, YKL-40, is overexpressed in asthma and correlates with clinical features (exacerbation, lack of control, FEV_1_). Cluster analyses have demonstrated that neutrophilic and obesity-related phenotypes are associated with the highest levels of YKL-40.

Although not specific to asthma, YKL-40 could have practical applications in the assessment of disease control and phenotype. Further studies would be useful to assess whether its monitoring could facilitate successful clinical decisions. Another area for future investigations is using YKL-40 as a target for novel anti-asthmatic therapies (59).

## Author contributions

KS: general concept of the manuscript and preparation of the manuscript. JR: preparation and correction of the manuscript and preparation of the figure. MN: correction of the manuscript. All authors have accepted the manuscript.
